# Mitigating effect of ferulic acid on di-(2-ethylhexyl) phthalate-induced neurocognitive dysfunction in male rats with a comprehensive in silico survey

**DOI:** 10.1007/s00210-023-02831-9

**Published:** 2023-11-15

**Authors:** Mhasen Khalifa, Rabie H. Fayed, Yasmine H. Ahmed, Ahmed A. Sedik, Nehad M. El-Dydamony, Heba M. A. Khalil

**Affiliations:** 1 Veterinary Hygiene and Management Department, Faculty of Vet. Medicine, Cairo University, Giza, 12211 Egypt; 2 Cytology and Histology Department, Faculty of Vet. Medicine, Cairo University, Giza, 12211 Egypt; 3 Pharmacology Department, Medical Research and Clinical Studies Institute, National Research Centre, Giza, 12622 Egypt; 4 Pharmaceutical Chemistry Department, College of Pharmaceutical Sciences and Drug Manufacturing, Misr University for Science and Technology, 6Th of October City, 12585 Egypt

**Keywords:** DEHP, Neurotoxicity, Ferulic acid, Oxidative stress, Cognition, Docking

## Abstract

Di-(2-ethylhexyl) phthalate (DEHP) is the most abundant phthalate threatening public health-induced neurotoxicity. This neurotoxicity is associated with behavioral and biochemical deficits in male rats. Our study investigated the neuroprotective effect of ferulic acid (FA) on male rats exposed to DEHP. Thirty-two male *Wistar* rats were assigned to four groups. Group I control rats received corn oil, group II intoxicated rats received 300 mg/kg of DEHP, group III received 300 mg/kg of DEHP + 50 mg/kg of FA, and group IV received 50 mg/kg of FA, all agents administrated daily per os for 30 days. Anxiety-like behavior, spatial working memory, and recognition memory were assessed. Also, brain oxidative stress biomarkers, including brain malondialdehyde (MDA), reduced glutathione (GSH), nitric oxide (NO), superoxide dismutase (SOD), brain-derived neurotrophic factor (BDNF) as well as heme oxygenase-1 (HO-1) were measured. Moreover, brain histopathology examinations associated with immunohistochemistry determination of brain caspase-3 were also evaluated. Furthermore, docking simulation was adapted to understand the inhibitory role of FA on caspase-3 and NO synthase. Compared to DEHP-intoxicated rats, FA-treated rats displayed improved cognitive memory associated with a reduced anxious state. Also, the redox state was maintained with increased BNDF levels. These changes were confirmed by restoring the normal architecture of brain tissue and a decrement in the immunohistochemistry caspase-3. In conclusion, FA has potent antioxidant and antiapoptotic properties that confirm the neuroprotective activity of FA, with a possible prospect for its therapeutic capabilities and nutritional supplement value.

## Introduction

Over recent decades, plasticizers have been used increasingly in industrial products such as automobiles, textiles, electronics, food packaging, and toys (Chang et al. 2021). Among polyvinyl chloride plastic’s most extensively utilized plasticizers is di-2-ethylhexyl phthalate (DEHP) (Ahmadpour et al. 2021). It is the most ubiquitous phthalate compound detected in environmental and food samples (Liu et al. 2023). Also, DEHP is one of the most prevalent endocrine-disrupting chemicals (EDCs), which is of special consideration because over 98% of the US population has measurable levels of DEHP and its bioactive metabolites (Hauser and Calafat 2005; Zota et al. 2014). The Endocrine Society describes endocrine-disrupting substances (EDCs) as exogenous chemicals or mixtures of chemicals that interfere with any aspect of hormone activity (Zoeller et al. 2012). DEHP is contained in plastic materials without a chemical bond; hence, it can easily diffuse into the environment at high temperatures or upon contact with hydrophobic materials through different routes, including ingestion, inhalation, dermal contact, and even prenatally (Rocha et al. 2017; Prasad 2021). Consequently, DEHP is regarded as a ubiquitous environmental contaminant that poses risks to public health (Ito et al. 2019).

Multiple studies have reported that DEHP is associated with reproductive system dysfunction, developmental abnormalities, liver toxicity, and alterations in thyroid function (Cheon 2020; Mondal et al. 2022). Meanwhile, some researchers have reported a strong association of DEHP with neurocognitive disorders (Lin et al. 2015; Kim et al. 2017) and neurobehavioral abnormalities, such as anxiety and memory impairment (Barakat et al. 2018; Gascon et al. 2015). Furthermore, perinatal exposure to DEHP could cause autistic-like behaviors in newborns (Li et al. 2022) and attention-deficit/hyperactivity disorder in children (Praveena et al. 2020).

DEHP-induced neurotoxicity in male rats has been confirmed, as male mice treated with low and high doses of DEHP exhibited behavioral deficits, including spatial memory impairments and anxiety-like behavior, and these effects were associated with androgen receptor (AR) downregulation in the hippocampus (Barakat et al. 2018).In addition, DEHP is regarded as gender-specific because male mice, but not female mice, treated with DEHP exhibit spatial learning and memory deficiencies (Zhao et al. 2020). Further, DEHP has a detrimental influence on hippocampus development in male rats but not in female rats, as revealed by Smith et al. (2011, 2015). Furthermore, male rats were sensitive to multiple doses of DEHP, as evidenced by an increase in Morris water maze (MWM) escape latency in males exposed to low and high doses of DEHP. However, females subjected to high doses of DEHP exhibited impaired working and spatial memory (Safarpour et al. 2021). Also, postnatal exposure to DEHP did not affect the hippocampus of female rats (Smith et al. 2011, 2015; Smith and Holahan 2014), indicating that males might be more susceptible to the neurotoxicity of DEHP.

Oxidative stress is characterized by an imbalance between the antioxidant system and pro-oxidant system resulting increase of reactive oxygen species (ROS) and reactive nitrogen species (RNS), which are considered oxidizing compounds (Sies et al. 2017). Multiple investigations have proven that oxidative stress triggers neurodegenerative disorders (Garcez et al. 2022; Santos et al. 2013). Furthermore, DEHP-induced neurotoxicity was linked with oxidative stress in the brain via an increment in MDA and intracellular ROS and a reduction in SOD and GSH brain levels (Wu et al. 2019; Safarpour et al. 2021).

Ferulic acid (FA) or (4-hydroxy-3methoxyphenyl) is a polyphenolic compound extracted from *Ferula asafoetida*. It can be found in fruits and vegetables, including broccoli, grapefruit, oranges, cereal bran, and beverages like coffee and beer (Silva and Pogačnik 2020). Also, it is considered a powerful antioxidant that can scavenge hydrogen peroxide, hydroxyl radicals, superoxide radicals, and nitroso peroxide (Xie et al. 2020). Moreover, FA can cross the blood–brain barrier (BBB) and reach the hippocampus (Zhang et al. 2011). Also, it inhibits neuroinflammatory responses and promotes hippocampal neurogenesis, exhibiting antiapoptotic, antitumor, and antithrombosis properties (Kaur et al. 2022). Furthermore, FA was reported to increase neuronal survival (Kaur et al. 2018) and has a protective function in several neurodegenerative diseases, such as Alzheimer’s disease (AD) and Parkinson’s (Di Meo et al. 2020), as well as improving cognitive impairment (Hassanzadeh et al. 2017; Wang et al. 2017).

Extensive research has shown that the heme oxygenase/biliverdin reductase system (HO/BVR) has a critical role in the central nervous system (CNS) (Mancuso and Santangelo 2014). It presents in two main forms, namely HO-1 and HO-2, and is responsible for enhancing cell stress response. Moreover, the deregulation of the HO-1 system was associated with several neurodegenerative disorders, neurotoxicity, and neuroinflammation (Nitti et al. 2018). Mhillaj and his colleagues shed light on the implication of HO/BVR in improving the neurocognitive profile of FA-exposed rats (Mhillaj et al. 2018). Also, Catino and his colleagues investigate the regulatory role of FA against trimethyltin-induced neuronal damage in the human neuroblastoma cell line SH-SY5Y via the Nrf2/HO-1 system (Catino et al. 2015). Therefore, FA may be acting through upregulating the HO/BVR system.

Herein, in our study, we investigate the therapeutic effect of FA and its capability to mitigate neurocognitive impairment and anxiety-like behavior induced by DEHP in male rats. That was achieved by measuring brain oxidative stress biomarkers, brain-derived neurotrophic factor (BDNF), and HO-1 levels with an assessment of brain histopathology and determining the immunohistochemical caspase-3 as an apoptotic marker of brain tissue. Moreover*,* functional and structural alterations of macromolecules within brain tissue cells were assessed using Fourier transforms infrared spectroscopy (FTIR). Also, in silico simulation was adapted to detect the pharmacokinetic behavior of the FA using ADMET and toxicity studies. Moreover, docking simulation was utilized to understand FA’s binding affinity and inhibitory role on caspase-3 and nitric oxide synthase (NO).

## Materials and methods

### Chemicals

Di(2-ethylhexyl) phthalate (C_24_H_38_O_4)_ (DEHP, CAS No. 117–817; purity ≥ 99) and FA C10H10O4 (FA Cat. No. 12,870–8) were purchased from Sigma Chemicals CO (ST. Louis, MO). BDNF was evaluated in brain homogenate using an ELISA kit (catalog no. BSKR60236-96 T) purchased from Boster Biological Technology, CO. California. Levels of GSH in brain homogenate were evaluated using Biodiagnostic Egypt’s kits (Catalog No. GR 25 11). Levels of MDA in brain homogenate were evaluated using Biodiagnostic, Egypt’s kits (Catalog No. MD 25 29). SOD was evaluated in brain homogenate using Biodiagnostic, Egypt’s kits (Catalog No. SD 25 21). NO was measured in brain homogenates using Biodiagnostic Egypt’s kits (Catalog No. NO 25 33). HO-1 was measured using MyBioSource Inc kit (USA, Catalog No. MBS2508238).

### Experimental design

#### Animals

Male *Wistar* rats were purchased from VACCERA in Egypt, weighing between 120 and 150 g with an average age of 2 months. They were acclimated for a week in a shoebox-shaped plastic cage with a 12-h light/dark cycle, 50% humidity, and a constant temperature of 25 ± 2 °C. The rodents had unlimited access to food and water.

#### Treatment groups

**Fig. 1:**
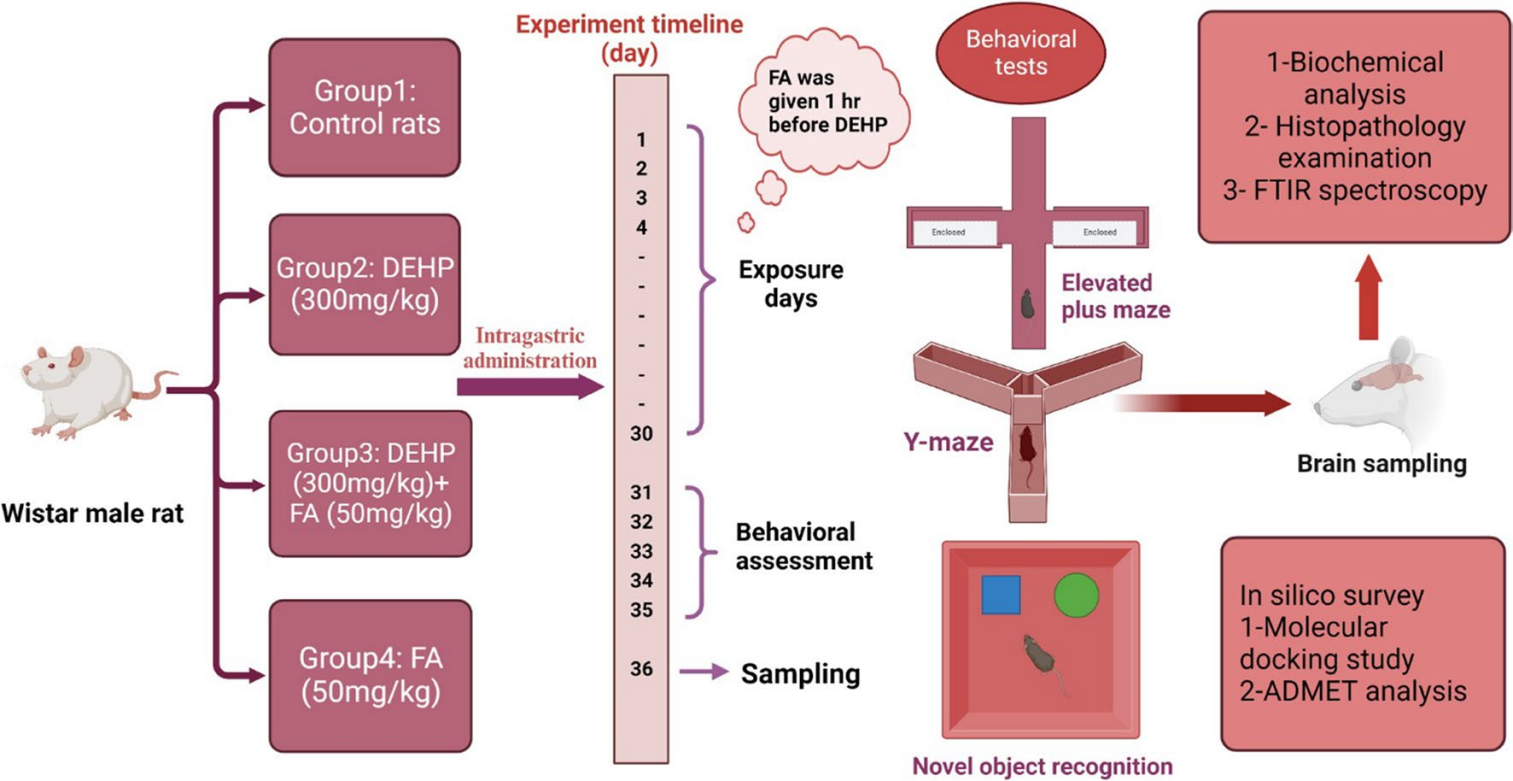
Schematic diagram for the experimental design. This study was conducted for 30 days and divided into four groups, including group 1: control rats received corn oil, group 2: DEHP (300 mg/kg), group 3: DEHP (300 mg/kg) + FA (50 mg/kg), and group 4: (50 mg/kg). All agents were given per os, with FA administered 1 h before DEHP. All groups were subjected to behavioral assessments 24 h after the last dose from the 31st to the 35th. Anxiety-like behavior, spatial working memory, and long-term memory were measured using elevated plus maze, Y-maze, and novel object recognition, respectively. On the 36^th^, brain sampling was collected for biochemical analysis, histopathological examination, and FTIR spectroscopy. Furthermore, in silico survey was conducted, such as a molecular docking study and ADMET analysis

Thirty-two male *Wistar* rats were randomly divided into four groups (each of 8 rats). Group 1: control rats received 2 ml/kg corn oil (DEHP and FA dissolvent); group 2: rats orally administered with 300 mg/kg bw of DEHP dissolved in 0.1 ml corn oil per 20 mg body weight (Fu et al. 2019; Wang et al. 2021) as toxic dose selection is based on previous research (Fu et al. 2018; Ran et al. 2019; Safarpour et al. 2021). Also, this dose of DEHP is reported by the European Union and the USA that stated DEHP is not harmful to the human at a dose of 48 mg/kg/day; the rat dose is equivalent to 300 mg/kg/day according to the surface area (Dong et al. 2019). Group 3: rats received 300 mg/kg bw of DEHP + 50 mg/kg bw of FA provided that FA was administrated 1 h before DEHP (El Morsy and Ahmed 2020). Group 4 rats administrated 50 mg/kg bw of FA in corn oil. The therapeutic dosage was selected based on previous research (Yu et al. 2021; Park et al. 2022). All the corresponding drugs were administrated daily via oral gavage starting at 9:00 a.m. (6 days per week) for 30 days. The experimental design is illustrated in Fig. 1.

### Behavioral assessment

Behavioral tests were conducted 24 h after the last dose of treatment. These tests started from the 31^st^ to the 35^th^ day. All behavioral test devices were cleaned with 70% ethyl alcohol to remove olfactory cues that may mislead our result. Also, they were allowed to dry from alcohol for 1 min between each test session. All behavioral tests were carried out manually by an experienced observer blinded to the experimental treatments.

#### Elevated plus maze

Elevated plus maze (EPM) is used to evaluate anxiety-like behavior in rodent animal models (Mohammad Abu-Taweel and Al-Fifi 2021). It depends on the interaction between fear and curiosity exhibited by rodents when exposed to a novel but threatening environment (Raony et al. 2021). The test was carried out in a wooden elevated maze consisting of two open arms and two closed arms 50 × 10 × 30 cm and 60-cm height from the floor, and the central platform united the four arms (Khalil et al. 2021). The rat was placed in the central platform facing the open arm for 5 min, and the time spent in the closed arms, the number of entries to closed arms, the time spent in the open arms, and the number of entries to open arms were recorded. When four paws are observed in the arm, we reveal that a rat has entered the arm.

#### Y-maze

Y-maze test assessed working spatial and short-term memory (Ekundayo et al. 2022) based on an animal’s natural tendency to explore new environments (Ikram et al. 2019). The test was conducted on a wooden apparatus consisting of three similar arms (120°, 40 cm long, and 35-cm height) identified as A, B, and C. In brief, each rat was randomly put at the end of one arm and allowed to move freely in all three arms for 5 min. The number of arm entries and spontaneous alternation percentage (SAP) were calculated according to this formula:

$$number\;of\;alternation/\left(total\;number\;of\;arm entries-2\right)\times100.$$

#### Novel object recognition test

Novel object recognition test (NOR) is based on animal curiosity to discover new objects and displayed memory performance and hippocampal function (Prakash et al. 2013; Lueptow 2017). This test was conducted over three session days, i.e., habituation, training, and testing with 24-h intervals (Zavala-Ocampo et al. 2024). The first day was the habituation phase in which the rat was put into a wooden open-field arena without objects (75 × 75 cm base, 40-cm height) and allowed to explore freely for 5 min. Then, through the training phase, rat was allowed to explore two identical objects for 5 min. Next, through the testing phase, rat was presented with two different objects, one old object, and one novel object, for 5 min. The preference for novel object% and discrimination ratio was calculated according to the following formula:

$$The\;preference\;for\;novel\;object\%=novel\;object\;exploration\;time/total\;exploration\;time\times100$$

$$DR=\left(novel\;object\;exploration\;time-old\;object\;exploration\;time\right)/\left(novel\;object\;exploration\;time+old\;object\;exploration\;time\right)$$

### Euthanasia and sampling

After the behavioral tests, all rats were euthanized by cervical dislocation, and the brain was excised rapidly and washed with cold saline to remove any blood clots. Halves of the brains were preserved in 10% neutral buffer formalin for histological and immunohistochemical analysis, and the other halves were preserved at – 80 °C for the subsequent biochemical analysis.

### Biochemical analysis

#### Evaluation of the BDNF levels using ELISA

BDNF was evaluated in brain homogenate. Ninety-six-well microtiter plates were pre-coated with an antibody specific to BDNF. After that, samples or standards were added to the plate wells prepared with a biotin-conjugated antibody specific to BDNF. Then, avidin conjugated to horseradish peroxidase (HRP) was added and incubated in each microplate well. Only the wells containing BDNF, biotin-conjugated antibody, and enzyme-conjugated avidin will change color after adding the TMB substrate solution. The enzyme–substrate reaction was stopped by adding a sulfuric acid solution, and the color change was measured spectrophotometrically at 450 ± 10 nm. The concentration of BDNF in the samples was determined by comparing their optical density (OD) to the standard curve (Elfving et al. 2010).

#### Evaluation of reduced glutathione levels in the brain

The brain levels of reduced glutathione (GSH) was assessed based on the reduction of 5,5′ dithiobis (2-nitrobenzoic acid) (DTNB) with GSH to produce a yellow compound. At 450 nm, the absorbance of reduced chromogen was calculated and was directly proportional to the concentration of GSH present (Jollow et al. 1974).

#### Evaluation of malondialdehyde levels in the brain

Levels of malondialdehyde (MDA) were evaluated based on the following principle: thiobarbituric acid (TBA) reacts with MDA in an acidic medium at 95 °C for 30 min to form a TBA acid reactive product, the absorbance of which can be measured at 534 nm (Ohkawa et al. 1979).

#### Evaluation of superoxide dismutase levels in the brain

The technique of superoxide dismutase (SOD) evaluation is based on the xanthine/xanthine oxidase system, which is a superoxide generator that inhibits nitroblue tetrazolium (NBT) reduction. After adding 1.0 ml of ethanol/chloroform mixture (5:3, v/v) to the 1.0 ml of sample and centrifuging, activity was evaluated in the ethanol phase of the supernatant. The amount of enzyme causing a 50% inhibition in the rate of NBT reduction was used to define one unit of SOD. The activity was expressed as units per mg of protein (Marklund and Marklund 1974).

#### Evaluation of NO levels in the brain

NO evaluation was performed in brain homogenate. Initially, nitrate was converted into nitrite by the enzyme nitrate reductase, and then, nitrite was quantified using Griess reagent at 550 nm, as previously described (Montogomery and Dymock 1961). Total nitrate plus nitrite (NO_3_ + NO_2_), the stable end products of NO metabolism, were used to measure NO. The concentration of NO in the homogenate was expressed as nmol/g tissue.

#### Evaluation of HO-1 levels in brain

HO-1 was measured by spectrophotometry at 450 nm, and HO-1 concentration was expressed as ng/mg tissue.

### Histopathological examination

#### Light microscopy

The brain fixed samples were dehydrated, followed by xylene, and embedded in paraffin. Sections 4 μm thick were prepared, deparaffinized, and stained with hematoxylin and eosin (H&E) for histopathological examination and immunohistochemistry (Bancroft and Gamble 2008).

#### Immunohistochemical examination

Caspase-3 as an apoptotic marker. The avidin–biotin-peroxidase technique (Hsu et al. 1981) detected activated caspase-3 as an apoptotic marker. Brown cytoplasmic or nuclear staining is considered a positive reaction. After deparaffinization and rehydration of the cerebellar sections, antigen retrieval was achieved by boiling sections in citrate buffer in a microwave. Endogenous peroxidase was blocked using H_2_O_2_. After blocking of non-specific background with 10% serum-tris buffer for 20 min at room temperature, the sections were then incubated with the primary antibody anti-caspase-3 rabbit polyclonal antibody (Catalog No. RB-1197 from Thermo-Fisher Scientific) diluted 1/100 at room temperature for 120 min. The slides were subsequently incubated with biotinylated polyvalent secondary antibody and then incubated with avidin–biotin-peroxidase complex solution (LSAB2 Kit; Dako). The reaction was visualized by adding 3,3′-diaminobenzidine tetrachloride to the sections. Hematoxylin was used to counterstain the sections. Slides stained with secondary antibody IgG only were used as negative controls. Specimens from palatine tonsils were used as positive controls.

#### Quantitative analysis of caspase-3 immunohistostaining

Five fields per section were chosen to measure the proportion of the total area percentage stained with the anti-caspase-3 antibody using the software Leica Quin 500 analyzer computer system (Leica Microsystems, Switzerland) at the Faculty of Dentistry, Cairo University. Mean values and SDs were obtained for each specimen and analyzed statistically.

### FTIR spectroscopy

Brain tissue samples were subjected to lyophilization with the aid of a labconco freeze dryer (Catalog No. 7754030, USA) for 24 h at − 50 °C. Lyophilized brain sections were mixed gently with potassium bromide (KBr) crystals under 1200 psi for 8 min to produce KBr pellets. For each rat, the IR spectra were retrieved from different KBr disks and then coadded. Pellets were scanned at 4 cm^−1^ at room temperature within the mid-IR spectra (3200–400 cm^−1^). The IR spectra were recorded using a PerkinElmer Spectrum (PerkinElmer Inc., USA). All IR spectra from each group were coadded, and each group was represented by one spectrum. The entire spectra were normalized, and the baseline was corrected by using IR solution software (Moll 1971).

### In silico* survey*

#### Molecular docking study

Molecular docking was done according to the reported procedures (Abdelnaby et al. 2022; El-Dydamony et al. 2022) using Discovery Studio 4.1 software (Accelrys, Inc., San Diego, CA, USA). The X-ray 3D structures of caspase-3 (PDB ID 1RHR) (Gadelmawla et al. 2022) and NO synthase (PDB ID 4NOS) (Fischmann et al. 1999) were obtained from the PDB site (http://www.rscb.org/pdb) and prepared for docking by cleaning the protein and fixing missing chains. The CHARMm forcefield was applied, and energy was minimized. The binding pockets were defined, and the validation step was done following the reported steps. Then, the docking of the prepared ligands into the 3D structures of the proteins was carried out, assuming flexible ligand-rigid receptor docking using CDOCKER protocol. The best ten poses were studied, and the one with the best score and orientation was chosen. Our ligands were joined with the reported amino acid residues (Gadelmawla et al. 2022).

#### ADMET analysis

The chemical structure of FA (PubChem CID 445858.) was retrieved from PubChem (http://pubchem.ncbi.nlm.nih.gov/) database and was submitted in the form of canonical SMILE to evaluate the pharmacokinetic properties and toxicity of FA by using the pkCSM online tool and the ProTox online tool (Banerjee et al. 2018; Pires et al. 2015), respectively.

### Statistical analysis

All quantitative results were analyzed using GraphPad Prism 9.4.1 (GraphPad Software, San Diego, CA). Data were presented as mean ± standard error (SEM). The experimental study was performed in multiple cohorts; therefore, comparisons among multiple group means were performed using a one-way analysis of variance, followed by Tukey’s post hoc test. Statistical significance was set at *P* ≤ 0.05.

## Results

### FA reduces DEHP-induced anxiety-like behavior in male rats

**Fig. 2:**
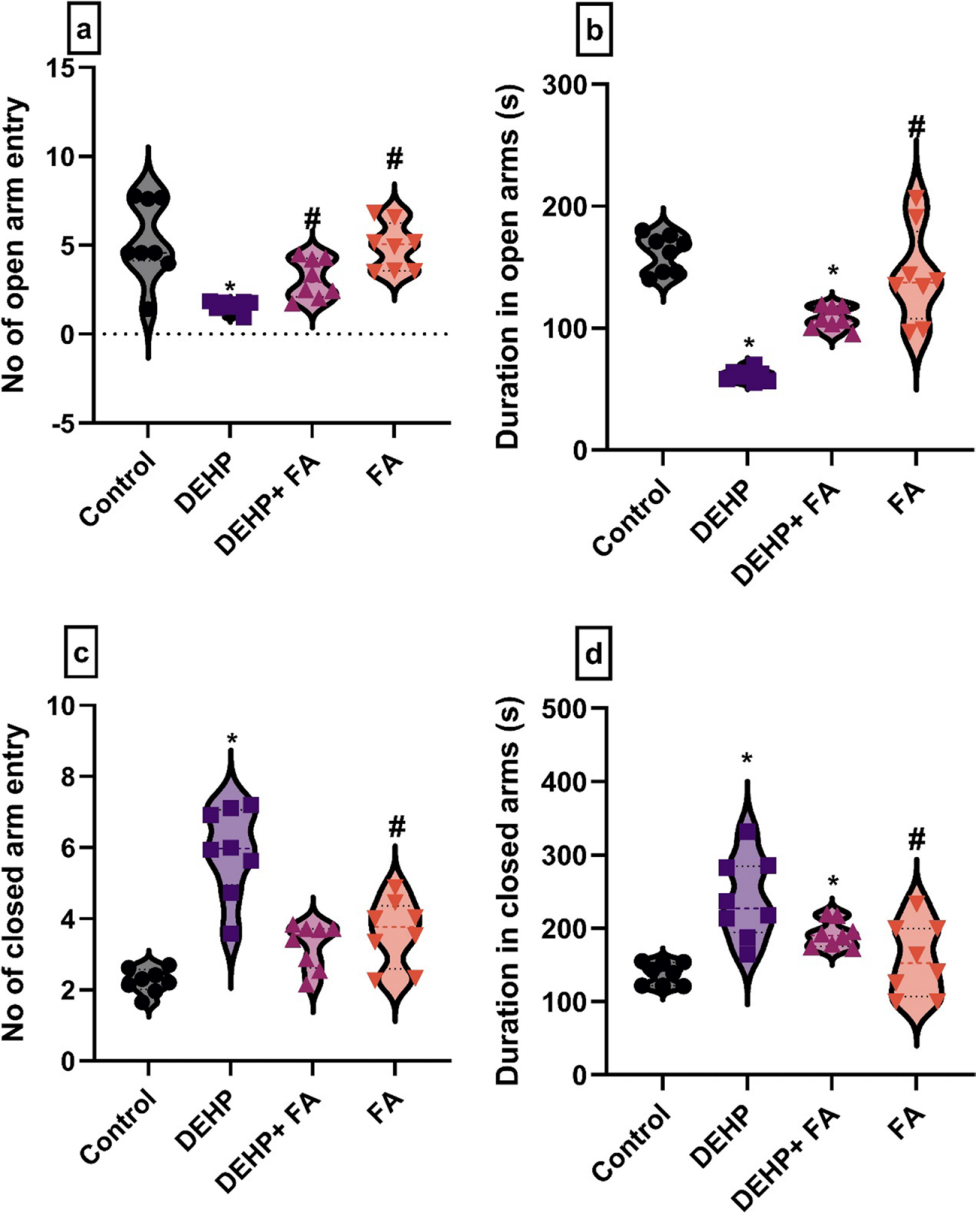
Effect of phthalates (DEHP) and ferulic acid (FA) administration on the anxiety-like behavior of male rats. **a** Elevated plus maze: number of open-arm entry. **b** Elevated plus maze: duration in open arm. **c** Elevated plus maze: number of closed-arm entry. **d** Elevated plus maze: duration in the closed arm. Data are expressed as mean ± standard error (SE) (one-way analysis of variance (one-way ANOVA) followed by Tuckey post hoc test for eight rats in each group. *Significantly different from the control group, *P* < 0.05. # Significantly different from the DEHP group, *P* < 0.05

DEHP-intoxicated rats exhibited anxiety-like behavior in the EPM test visualized by a significantly decreased number of open-arm entries (*F*(3, 28) = 11, *P* = 0.001) (1.66 ± 0.82, *P* < 0.05) with decreased duration in open arms (*F*(3, 28) = 34, *P* = 0.001) (60.33 ± 22.06, *P* < 0.05) associated with a significantly increased number of closed-arm entries (*F*(3, 28) = 25, *P* = 0.001) (5.66 ± 0.81, *P* < 0.05) with increased duration in closed arms (*F*(3, 28) = 10, *p* = 0.001) (239.66 ± 22.06, *P* < 0.05) compared to the control group and FA-treated group (Fig. 2a–d). Furthermore, the DEHP + FA-treated group showed a marked increase in the number of open-arm entries (3.33 ± 0.52, *P* < 0.05) with no significant difference in the number of closed-arm entries (3.5 ± 0.5, *P* > 0.05) compared to the DEHP group. Meanwhile, there was a significant decrease in the duration inside the open arms (108 ± 48, *P* < 0.05) with a substantial increase in the closed-arm duration (192 ± 48, *P* < 0.05) compared to the control group.

### FA enhances impairments in spatial working memory and recognition memory induced by DEHP in male rats

**Fig. 3:**
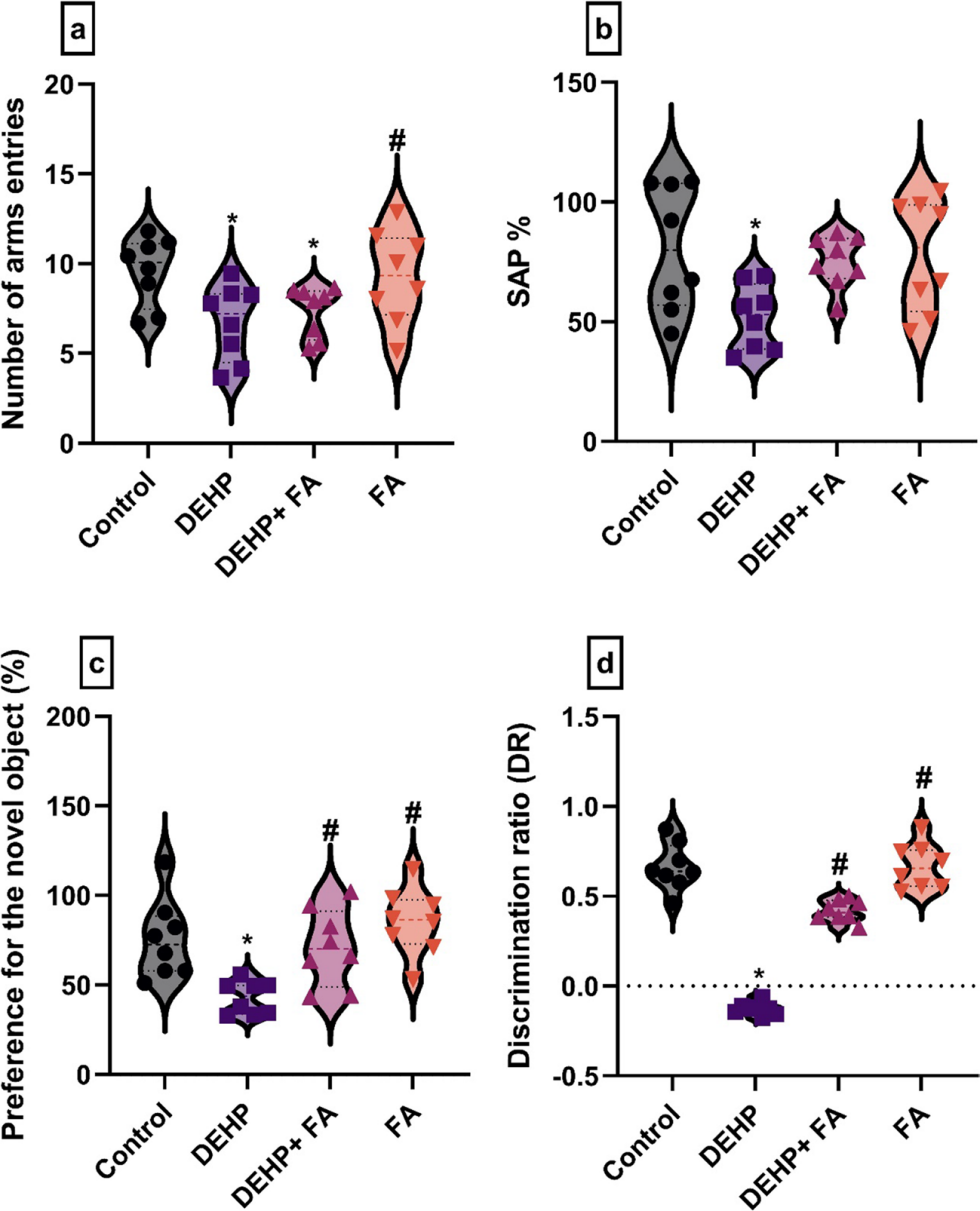
Effect of DEHP and FA administration on the spatial working memory and recognition memory of male rats. **a** Y-maze test: number of arm entries. **b** Y-maze test: spontaneous alternation percentage (SAP). **c** Novel object recognition test: preference for the novel object. **d** Novel object recognition test: discrimination ratio. Data are expressed as mean ± standard error (SE) using one-way analysis of variance (one-way ANOVA) followed by Tuckey post hoc test for eight rats in each group. *Significantly different from the control group, *P* < 0.05. #Significantly different from the DEHP group, *P* < 0.05

In the Y-maze test, one-way ANOVA showed significant difference between groups in the number of arm entries (*F*(3, 28) = 3.8, *P* = 0.02) and SAP (*F*(3, 28) = 4.1, *P* = 0.002). DEHP-intoxicated rats displayed a marked decreased number of arms entries (6.66 ± 0.50, *P* < 0.05) with a significant decline in the SAP (52.12 ± 16.96, *P* < 0.05) compared to control rats. Conversely, there was no significant difference in the SAP between the control, DEHP + FA, and FA groups (79.92 ± 19.28, 75 ± 19, 78.60 ± 15.70, *P* < 0.05, respectively) (Fig. 3a, b).

Also, in the NOR test, one-way ANOVA revealed marked difference between groups in the preference for the novel object percentage (*F*(3, 28) = 7.8, *P* = 0.001) and in the the discrimination ratio (*F*(3, 28) = 120, *P* = 0.001). DEHP group showed a significantly diminished preference for the novel object percentage (43.91 ± 10.92, *P* < 0.05) and a significantly reduced discrimination ratio (− 0.12 ± 0.21, *P* < 0.05) compared to the control group. Interestingly, the DEHP + FA group and the FA group showed a significant improvement in the discrimination ratio (0.42 ± 0.29 and 0.67 ± 0.22, *P* < 0.05, respectively) with a markedly elevated preference for the novel object percentage (71.83 ± 14.47, and 86.93 ± 12.16, *P* < 0.05, respectively) compared to DEHP intoxicated rats (Fig. 3c, d).

### Activity of FA on brain oxidative stress biomarkers, BNDF, and HO-1 levels in DEHP-intoxicated male rats

**Fig. 4:**
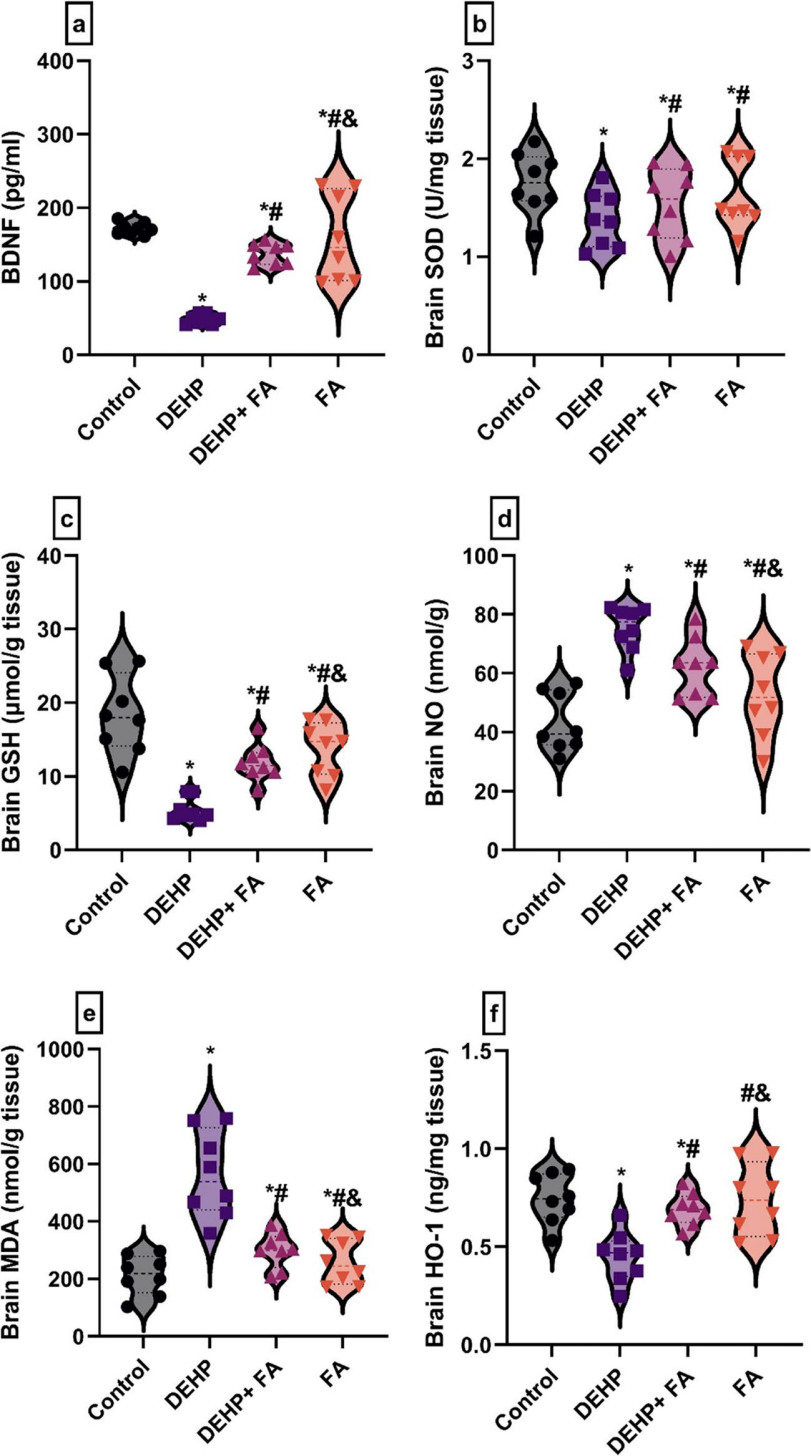
Effect of DEHP and FA administration on oxidative stress marker level in the male rat’s brain. **a** Brain-derived neurotrophic factor (BDNF). **b** Superoxide dismutase (SOD). **c** Reduced glutathione (GSH). **d** Nitric oxide (NO). **e** Malondialdehyde (MDA). **f** Heme oxygenase-1 (HO-1). Data are expressed as mean ± standard error (SE) using one-way analysis of variance (one-way ANOVA) followed by Tuckey post hoc test for eight rats in each group. *Significantly different from the control group, *P* < 0.05. #Significantly different from the DEHP group, *P* < 0.05. &Significantly different from the DEHP + FA group,* P* < 0.05

One-way ANOVA demonstrated a substantial difference between groups in the brain levels of BDNF (*F*(3, 28) = 26, *P* = 0.001), SOD (*F*(3, 28) = 1.9, *P* = 0.001), GSH (*F*(3, 28) = 13, *P* = 0.001), MDA (*F*(3, 28) = 22, *P* = 0.001), NO (*F*(3, 28) = 18, *P* = 0.001), and HO-1 (*F*(3, 28) = 8.7, *P* = 0.001). DEHP-intoxicated rats had significantly diminished BDNF (49 ± 1.47, *P* < 0.05), brain levels of SOD (1.37 ± 0.02, *P* < 0.05), and GSH (5.71 ± 0.89, *P* < 0.05) accompanied by a substantial increase in the brain MDA (561.7 ± 6.0, *P* < 0.05) and NO levels (75.31 ± 2.13, *P* < 0.05) compared to the control group. However, the DEHP + FA group showed a significant increment in the BDNF (137.8 ± 2.28, *P* < 0.05) as well as SOD (1.54 ± 0.01, *P* < 0.05) and GSH (11.7 ± 0.40, *P* < 0.05) with a substantial decrement in the brain MDA (298.3 ± 6.0, *P* < 0.05) and NO (62.31 ± 1.23, *P* < 0.05) compared to DEHP-intoxicated rats. On the other hand, the brain HO-1 levels were markedly decreased in the DEHP-intoxicated rats (0.43 ± 0.007, *P* < 0.05) compared to control rats (0.74 ± 0.004, *P* < 0.05). At the same time, the DEHP + FA group displayed a significant increase (0.73 ± 0.0, *P* < 0.05) in the brain HO-1 levels compared to DEHP-intoxicated rats (Fig. 4a–f).

### Histopathological investigations

#### Light microscopic observations

**Fig. 5:**
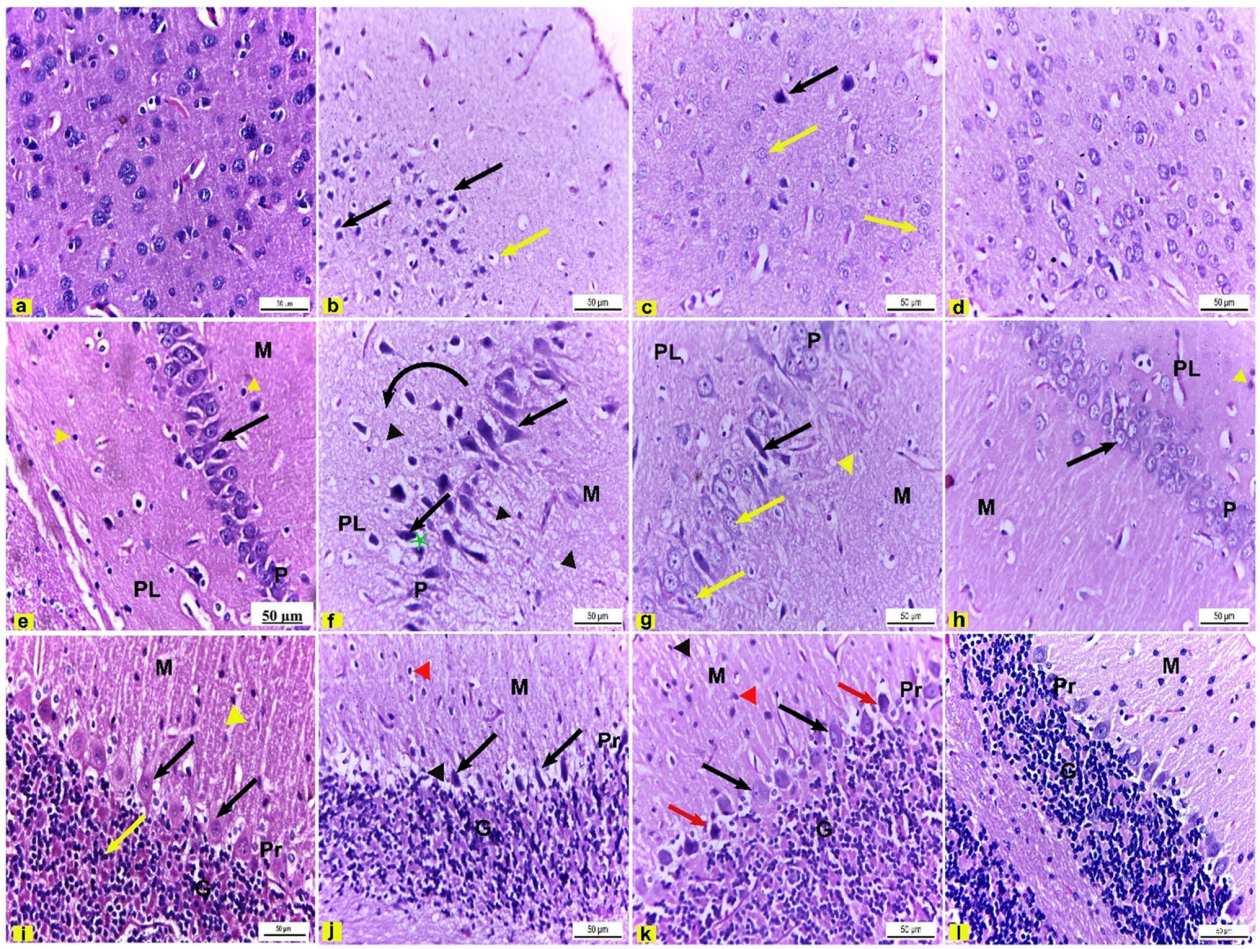
Brain sections of male albino rats. H&E × 400. **a** Cerebral cortex section of control rats showed normal structure and distribution of neurons and neuroglia on neuropil. **b** Cerebral cortex sections of DEHP-intoxicated rats revealed shrunken pyknotic pyramidal neurons (black arrow) and neuroglia (yellow arrow) with pericellular spaces. **c** Cerebral cortex of DEHP + FA rats revealed marked maintenance of normally shaped pyramidal neurons that had vesicular central nuclei (yellow arrows) and few neurons still pyknotic (black arrow). **d** FA-treated rats had cerebral cortex with normal structure and distribution of neurons and neuroglia on neuropil. **e** Hippocampus sections of control rats showed the normal structure of molecular layer (M) that consisted of neurons and neuroglia (yellow arrowhead), pyramidal cell layer (P) that formed of linear arranged triangular-shaped neurons with vesicular central nuclei (black arrow), and polymorphic layer (PL) formed of neurons and neuroglia (yellow arrowhead). **f** Hippocampus of DEHP-intoxicated rats revealed neuropil vacuolation (black arrowhead), degenerated and pyknotic pyramidal neurons (black arrow) with perineural spaces (green star), and disarrangement of triangular-shaped neurons (arched black arrow). **g** Hippocampus of DEHP + FA showed obvious recovery in the form of diminished neuropil vacuolation (yellow arrowhead), and many pyramidal neurons appeared nearly normal (yellow arrows) except for a few neurons that appeared pyknotic with pericellular space (black arrow). **h** FA-treated rats had normal hippocampus layers, molecular (M), pyramidal (P) with triangular-shaped neurons (black arrow), and polymorphic (PL) cell layers, respectively. **i** Cerebellar cortex of control rats consisted of molecular (M) that had stellate neurons (yellow arrowhead), Purkinje (P) that had pear-shaped neurons with large central vesicular nuclei (black arrows), and granular (G) cell layers, respectively**. j** Cerebellar cortex of DEHP-intoxicated rats revealed pyknotic and degenerated Purkinje cells (black arrow) with perineuronal spaces (black arrowhead) and pericellular spaces (red arrowhead) in the molecular layer. **k** Cerebellar cortex of DEHP + FA showed marked maintenance of normal pear-shaped Purkinje cells (black arrows); most of the stellate cells appeared nearly normal (red arrowhead), but few Purkinje cells (red arrows) and stellate cells (black arrowhead) still pyknotic with perineural space. **l** FA-treated rats had normal cerebellar cortex layers, respectively, molecular (M), Purkinje (P), and granular (G) cell layers

H&E-stained sections of the cerebral cortex of control rats showed normal histological structure and distribution of neurons and neuroglia on the neuropil (Fig. 5a). However, cerebral cortex sections of rats treated with DEHP revealed structural degenerative changes of neurons compared to the control group as the pyramidal neurons, and neuroglia appeared pyknotic and shrunken with pericellular spaces (Fig. 5b). Conversely, DEHP + FA group exhibited marked maintenance of cerebral cortex architecture compared to DEHP-intoxicated group evidenced by nearly normal appearance of pyramidal neurons that had vesicular central nuclei except some neurons still pyknotic with pericellular spaces (Fig. 5c). Moreover, cerebral cortex sections of FA-treated rats showed the normal structure of neurons and neuroglia same as control rats (Fig. 5d).

Hippocampus sections of control rats were composed of normal histological structures of three layers; molecular, pyramidal, and polymorphic cell layers, respectively. The molecular and polymorphic layers consisted of scattered neurons and neuroglia on the neuropil background. The pyramidal (principle) cell layer formed of a linear row of triangular-shaped neurons that had central vesicular and spherical nuclei with prominent nucleoli (Fig. 5e). Contrariwise, hippocampus sections of DEHP intoxicated rats revealed several histopathological alterations such as neuropil vacuolation in three layers, disarrangement of triangular-shaped neurons from its linear arrangement, and pyramidal neurons appeared degenerated and pyknotic with perineural spaces (Fig. 5f). On the other hand, DEHP + FA group showed obvious recovery in the form of diminishing the neuropil vacuolation, many pyramidal neurons appeared nearly normal with vesicular spherical nuclei, but few neurons still pyknotic with pericellular space (Fig. 5g). FA-treated rats had nearly normal histological structures of hippocampus layers: the molecular, pyramidal, and polymorphic cell layers, respectively, similar to control rats (Fig. 5h).

Histological examination of cerebellar cortex sections of control rats showed the normal structure of the three layers: molecular, Purkinje, and granular cell layers, respectively. The molecular layer consisted of small stellate neurons. The Purkinje cell layer is formed of pear-shaped neurons with large vesicular nuclei in a linear arrangement. Purkinje cells had T-shaped dendrites extended to the molecular layer. The granular layer had small dark nuclei of neurons (Fig. 5i). On the contrary, the cerebellar cortex of DEHP-intoxicated rats revealed degenerated and pyknotic Purkinje cells with pericellular spaces and perineural spaces in the molecular layer compared to the control group (Fig. 5j). However, cerebellar cortex of DEHP + FA-treated rats showed marked maintenance of normal pear-shaped Purkinje cells with vesicular nuclei. Most of the stellate cells appeared normal except for a few Purkinje, and stellate neurons appeared pyknotic with perineural spaces (Fig. 5k). Cerebellar cortex of FA-treated rats had the normal histological structure of the three layers of the same as the control group (Fig. 5l).

#### Immunohistochemical observations

**Fig. 6:**
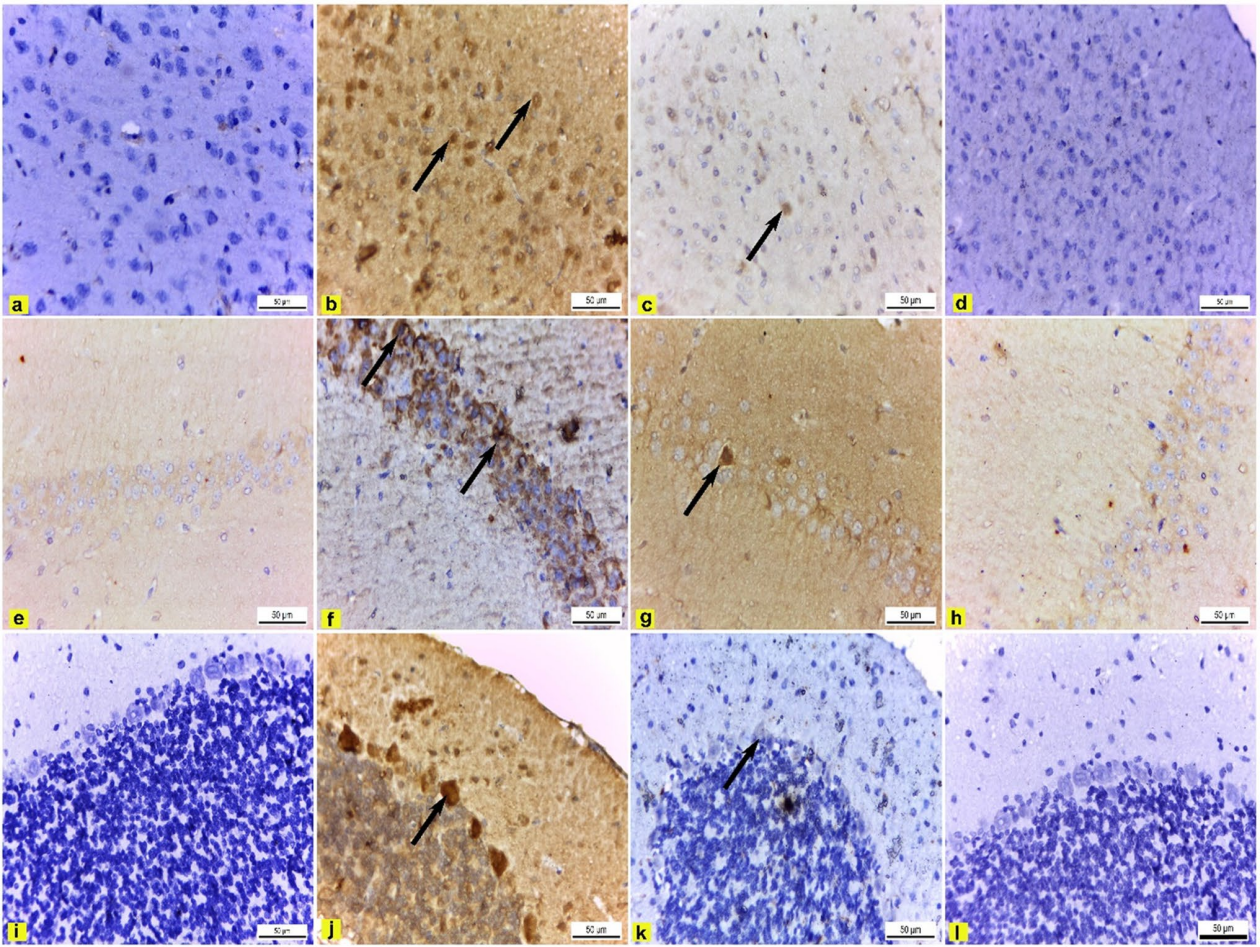
Immunohistochemical caspase-3-stained brain sections × 400. **a**, **e**, and **i** Control rats’ cerebral cortex, hippocampus, and cerebellar cortex had negative caspase-3 immunoexpression. **b**, **f**, and **j** DEHP-intoxicated rats’ cerebral cortex, hippocampus, and cerebellar cortex revealed strong positive caspase-3 immunoexpression (black arrow). **c**, **g**, and **k** DEHP + FA-treated rats’ cerebral cortex, hippocampus, and cerebellar cortex showed mild caspase-3 immunoreaction (black arrow). **d**, **h**, and **l** FA-treated rat’s cerebral cortex, hippocampus, and cerebellar cortex showed negative caspase-3 immunoexpression

**Fig. 7:**
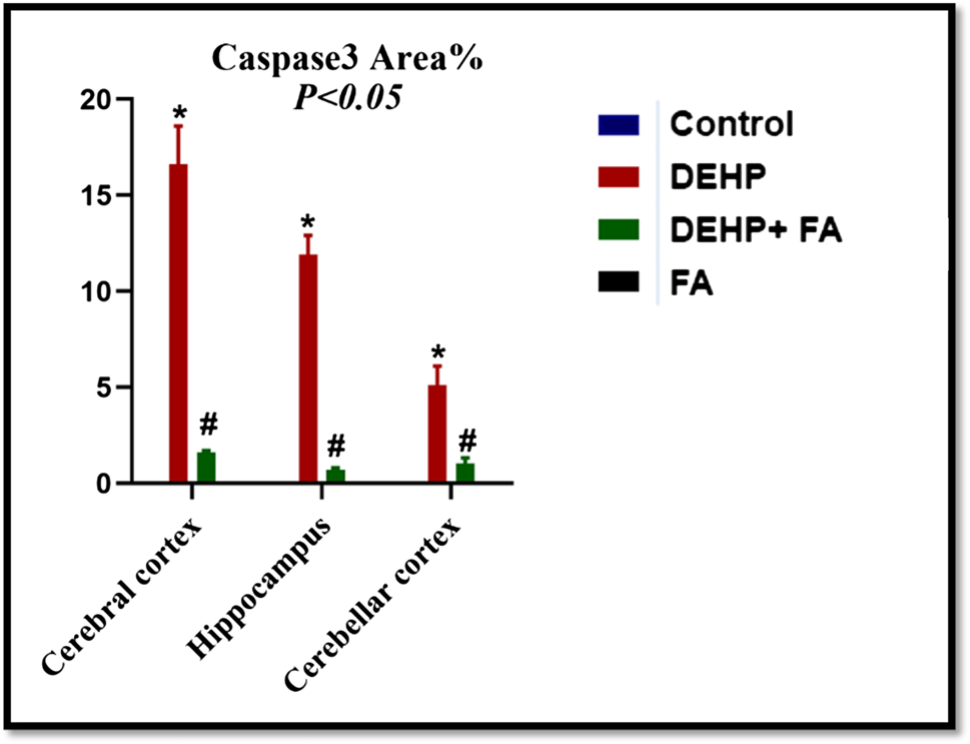
Effect of DEHP and FA administration on the percent area covered by caspase-3-positive immunoreactive cells within the brain of rats. The results were expressed as mean ± SE. *Significantly different from the control group. ^**#**^Significantly different from the DEHP-intoxicated group. *P* value ≤ 0.05

Immunohistochemical examination of the cerebral cortex, hippocampus, and cerebellar cortex sections of control rats showed negative caspase-3 immunoexpression (Figs. 6a, e, i and 7). On the contrary, cerebral cortex, hippocampus, and cerebellar cortex sections of DEHP-intoxicated rats revealed strong positive caspase-3 immunoreactivity that significantly increased by 16.6, 11.9, and 5.1, respectively, compared to control rats (Figs. 6b, f, j and 7). However, DEHP + FA-treated rats exhibited mild caspase-3 immunoreaction in the cerebral cortex, hippocampus, and cerebellar cortex sections that significantly reduced by 1.6, 0.7, and 1.03, respectively, compared to DEHP group (Figs. 6c, g, k and 7). Moreover, cerebral cortex, hippocampus, and cerebellar cortex sections of FA-treated rats showed negative caspase-3 immunoexpression similar to control rats (Figs. 6d, h, l and 7).

### FTIR spectroscopy

**Fig. 8:**
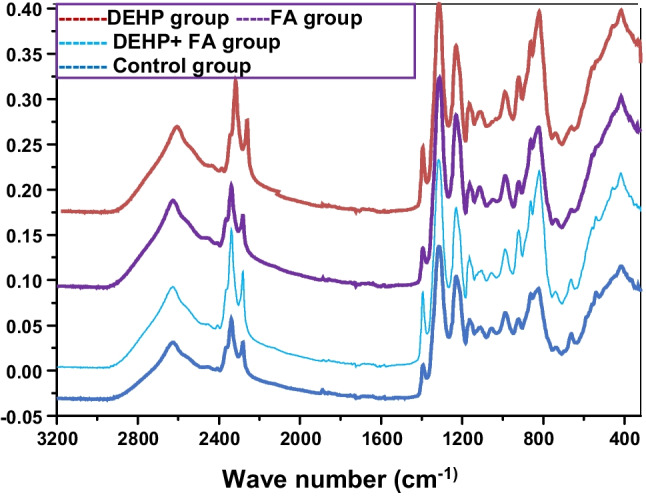
Effect of FA on wave number alterations in brain tissue of DEHP-intoxicated rats. Representative infrared spectra of lyophilized brain samples from different experimental groups

The FTIR spectra of various substances are shown in Fig. 8 and Table 1, and the wave number alterations in DEHP-intoxicated rats were 1.1 ± 0.003 compared to the control group 4.45 ± 0.002. While the value in the FA group was 3.70 ± 0.002 and in the DEHP + FA group was 4.25 ± 0.002. Interestingly, DEHP + FA returned the value to a normal value.

**Table 1:** Effect of FA on wave number alterations from FTIR spectra in the brain of rats intoxicated with DEHP

Groups	Control	DEHP	DEHP + FA	FA
Wave number alterations	4.44 ± 0.002	1.1 ± 00.003^*^	4.25 ± 0.002^**#**^	3.70 ± 0.002^**#**^

^*^Data are expressed as mean ± SE using one-way ANOVA followed by Tukey’s post hoc test

^*^Significantly different from the control group, *P* < 0.05

^#^Significantly different from the DHEP group, *P* < 0.05

### In silico* survey*

#### Docking simulation

**Fig. 9:**
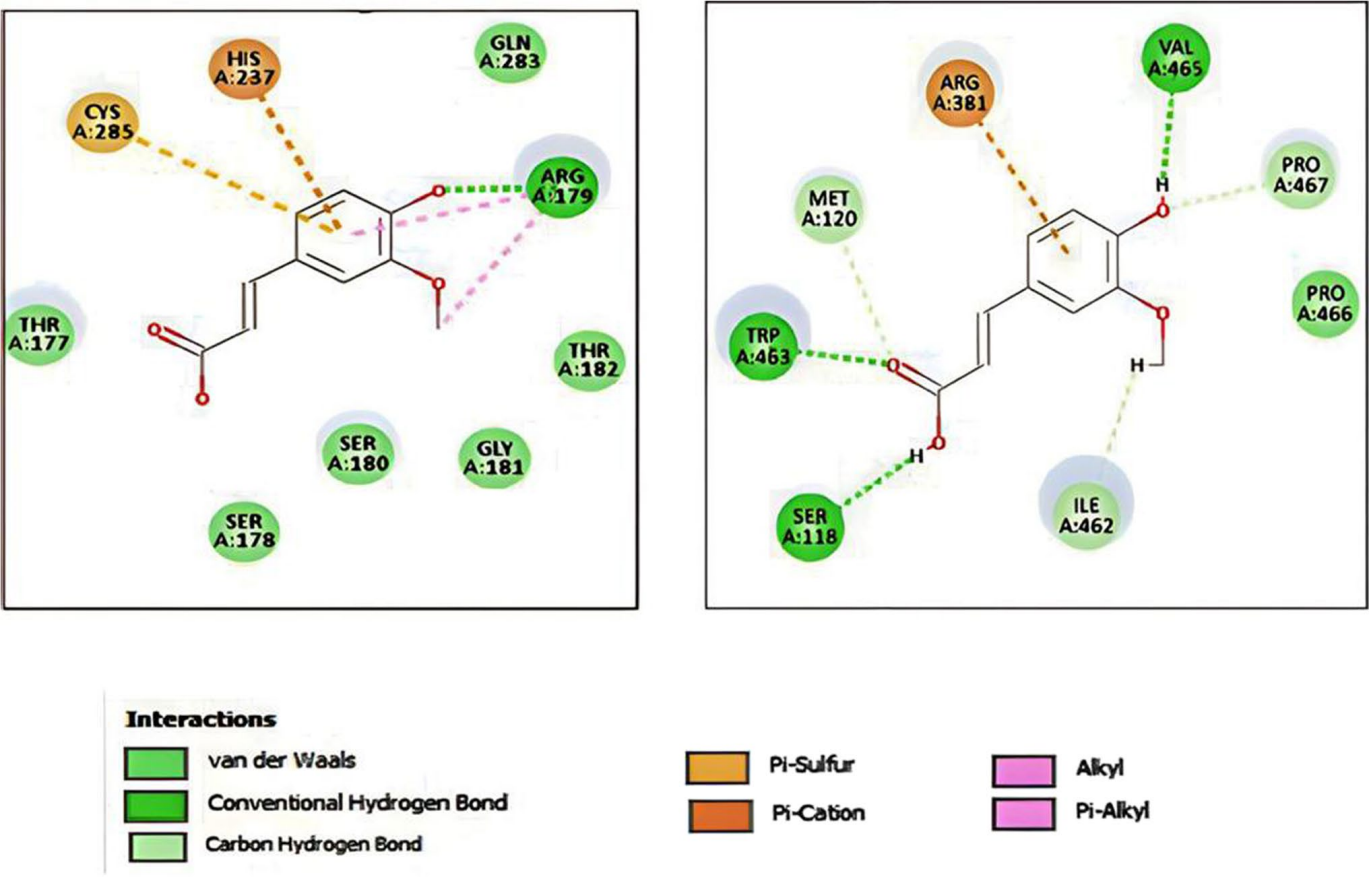
Two-dimensional interaction of FA with the active site of **a** caspase-3 and **b** NO synthase. **c** color codes for interactions

FA binding revealed one hydrogen bond with ARG179 through its phenolic OH and two alkyl interactions with the same amino acid residue (Fig. 9a). Also, HIS237 imparted a Pi-cation interaction, and CYS285 shared a Pi-sulfur interaction with the aromatic ring. On the other hand, docking of FA with NO synthase enzyme was performed to illustrate its inhibitory binding pattern. FA imparted three hydrogen bonding interactions: first: the phenolic OH assembled one hydrogen bond with VAL465 residue. Second, the carboxylic acid group shared two hydrogen bonds with SER118 and TRP463 through its OH and CO functionalities (Fig. 9b). MET120, PRO467, and ILE462 residues also exposed carbon hydrogen interactions.

#### ADMET analysis

As depicted in Table 2, the skin permeability of FA is − 2.720 cm/h, while the Caco-2 permeability is 0.176. The volume of FA distribution =  − 1.367, and it can penetrate the BBB moderately (− 0.239). Also, FA does not inhibit the CYP2D6 and CYP3A4 enzymes, and the log CLTOT value of the FA = 0.623 ml/min/kg. The ligand properties of FA displayed in Table 3 showed that no violation of any criteria for the Lipinski rule was detected.

**Table 2:** ADMET properties of ferulic acid by ProTox online tool

Toxicity			Excretion		Metabolism		Distribution			Absorption		
Class	LD50 (mg/kg)	Hepato toxicity	Ames toxicity	Total clearance	CYP3A4 inhibitor	CYP2D6 inhibitor	CNS	BBB	VDss	Caco-2	Skin	Intestinal
4	2.282	No	No	0.623	No	No	− 2.612	− 0.239	− 1.367	0.176	− 2.72	93.685

**Table 3:** Ligand properties of the FA

Molecular weight	194.186
Rotatable bonds	3
Log *P*	1.4986
Hydrogen bond acceptor	3
Hydrogen bond donor	2
Surface area	81.065

## Discussion

DEHP is a ubiquitous toxicant in the environment with nonbiodegradable nature, leading to persistent environmental contamination and exacerbating the public health burden (Kumar et al. 2021; Han et al. 2022). Previous studies reported that exposure to DEHP causes neurotoxicity and neurobehavior deficits (Kang et al. 2021; Nadeem et al. 2021; Yang et al. 2023). Therefore, our study investigates the neuroprotective effect of FA against neurotoxicity induced by DEHP. The current study demonstrates that FA therapy improves the behavior deficits of DEHP-intoxicated rats, including anxiety-like behavior, spatial working memory, and long-term memory. Also, brain oxidative stress biomarkers, BDNF, and HO-1 have been enhanced in the DEHP + FA group compared to DEHP-intoxicated rats. Moreover, FA has a high BBB penetrance, resulting in a neuroprotective effect against structural and functional alterations in lipid, protein, and nucleic acid levels in DEHP + FA rats compared to intoxicated rats, as shown in slico and FTIR spectroscopy. Further, FA restored the normal architecture of brain tissue with a significant decrease in caspase-3 expression in the DEHP + FA group compared to the DEHP-intoxicated rats.

Neurobehavioral deficits induced by DEHP were investigated using the EPM to evaluate anxiety-like behavior, Y-maze to assess spatial working memory, and NOR to measure long-term memory. DEHP-intoxicated rats exhibited anxiety-like behavior that was visualized by increased duration in the closed elevated arm with an increasing number of entries of the closed arm that indicates an anxious state of DEHP-intoxicated rats compared to other groups. Our results agree with previous studies (Barakat et al. 2018; Minatoya and Kishi 2021). In contrast, few studies have found that prenatal or adolescent male rats exposed to low doses of DEHP did not exhibit anxiety-like behavior (Sellinger et al. 2020; Khalifa et al. 2023). On the contrary, FA reversed the anxious state of DEHP-intoxicated rats, confirming the neuroprotective effect of FA and supported by a previous study (Deng et al. 2022).

Similarly, we evaluated rats’ spatial working and long-term memory using the Y-maze test and NOR. DEHP-intoxicated rats showed impairment in SAP that was visualized in the Y-maze test. While in the NOR, there is a decline in the discrimination ratio and preference for novel object %, indicating recognition and long-term memory deficit compared to control. These results agree with Ran et al. (2019) and Safarpour et al. (2021), which reported neurocognitive deficits in rats exposed to different doses of DEHP. On the contrary, the working memory of male and female pups exposed to low doses of DEHP was not significantly affected (Safarpour et al. 2021). Nevertheless, the administration of FA showed a significant improvement in the discrimination ratio and preference for the novel object %, which is crucial to long-term memory and learning ability. Our results agree with previous studies (Park et al. 2022; Wang et al. 2021).

Nitric oxide (NO) is a unique neuromodulator gasotransmitter implicated in multiple neurodegenerative illnesses, including Alzheimer’s disease (AD). NO is considered an endogenous molecule synthesized from L-arginine by the enzyme nitric oxide synthase (NOS), and several of NO’s central activities depend on NOS (Dubey et al. 2020). Further, NO is a crucial molecule for learning and memory since it modulates synaptic plasticity and long-term potentiation, which are vital steps for learning and memory (Domek-\Lopacińska and Strosznajder 2010). Also, NO produced by NOS or released from an endogenous S-nitrosothiol, GSNO, may upregulate the antioxidative thioredoxin system and the anti-apoptotic Bcl-2 protein via cGMP-dependent processes. NO has also been proven to have direct antioxidant effects by reacting with free radicals and iron-oxygen complexes (Dubey et al. 2018). Furthermore, oxidative stress is one of the critical mechanisms of DEHP-induced neurotoxicity, leading to neurobehavior impairments (Tang et al. 2015).

In our study, DEHP-intoxicated rats exhibited high lipid peroxidation levels (MDA) and brain NO, as well as a diminishment in GSH and SOD compared to other groups. Our results are in agreement with Safarpour et al. (2021). Conversely, FA-treated rats displayed a significant decline in levels of MDA and NO and significantly improved levels of GSH and SOD. These results are consistent with previous studies (Bao et al. 2019; Singh et al. 2021), which confirmed that FA possesses antioxidant properties and could inhibit oxidative stress. In addition, HO-1 upregulation is considered a natural defense mechanism against oxidative stress in brain tissue (Nitti et al. 2018). Herein, DEHP-intoxicated rats displayed reduced HO-1 levels compared to other groups. However, FA-treated rats exhibited a marked increase in HO-1 brain levels. This finding is in agreement with previous studies (Catino et al. 2015; Mhillaj et al. 2018).

Moreover, the neuroprotective action of HO-1 is not limited only to its antioxidant activity but also to the enhancement of neurotrophic factor production (Hung et al. 2010). BDNF is a protein belonging to the neurotrophin subfamily that has a crucial role in various aspects of CNS, including neural survival, synaptic plasticity, spatial working memory, and long-term memory (Lu et al. 2018). BDNF maintains neural survival and synaptic transmission, and its deficiency is associated with the pathophysiology of neuropsychiatric diseases (Smith and Holahan 2014). BDNF has been linked to the etiology of Alzheimer’s disease, and its expression is reduced in the hippocampus and several cortical regions of Alzheimer’s patients (Tian et al. 2014). In our study, DEHP-intoxicated rats significantly reduced BDNF levels compared to the control group. These findings are consistent with prior research confirming reduced levels of BDNF in neurotoxicity rat models induced by other toxicants (Abbas et al. 2022; Lee et al. 2022). Moreover, our study agrees with Smith and Holahan (2014), who reported that postnatal administration of DEHP to male rats decreases the expression of BDNF mRNA in the hippocampus by an average of 50% compared to control male rats. Also, it has been observed that prenatal toxicity by DEHP induces spatial learning and working memory deficits associated with the reduction of BDNF levels in either the hippocampus or the prefrontal cortex, which are recovered by exercise training during childhood-adolescence in male rats (Wang et al. 2020; Sun et al. 2021). However, FA administration showed an improvement in BDNF levels. These results are in agreement with Sasaki et al. (2019) and Nakayama et al. (2020).

Several studies have stated the significance of BDNF-NO interactions and established that both molecules have similar biological effects on neurons, such as in multiple neuronal systems’ growth, development, and functionality (Tian et al. 2014). It has been reported that endogenous NO regulates BDNF production. Moreover, BDNF stimulates neuronal NOS (nNOS) gene expression and NO generation, while NO synthesis elevates BDNF levels (Cheng et al. 2003; Chen et al. 2006). Additionally, NO enhances BDNF levels, stimulating neurogenesis in the hippocampus and improving memory (Lee et al. 2002). Our findings are consistent with Beheshti et al. (2021), who found scopolamine-induced learning and memory impairment by disrupting oxidative stress redox such as increased NO, promoting neuroinflammation, and decreasing BDNF, which was mitigated by the renin-angiotensin system (RAS). Furthermore, L-arginine, a precursor of NO, alleviated cognitive impairments, potentially due to a NO-mediated improvement in insulin sensitivity in type 2 diabetic mellitus (T2DM)-induced Alzheimer’s disease (AD) (Miczke et al. 2015; H. Dubey et al. 2022a, b). Similar studies supported our results, such as Memarpour et al. (2020) and Harikesh Dubey et al. (2022a, b).

Moreover, these changes were confirmed by several histopathological degenerative changes in all regions of the brain of DEHP-intoxicated rats: the cerebral cortex, hippocampus, and cerebellar cortex in the form of pyknotic neurons and neuroglia with pericellular spaces and neuropil vacuolation. Disarrangement of pyramidal neurons from its linear arrangement was observed in the hippocampus. Our findings agreed with a previous study (Basha and Radha 2020), which reported shrunken and darkened pyramidal cells of the hippocampus CA1 region of rats orally gavaged di-n-butyl phthalate. Moreover, our results were supported by Ma et al. (2015), who stated that the hippocampus of rats treated with diisononyl phthalate revealed disordered arrangements of neurons and deformations of cells. Due to its chemical structure, DEHP-induced neurotoxicity may be attributed to its ability to pass through the blood–brain barrier, causing an increase in excitatory and decreased inhibitory amino acids (Seif et al. 2017). On the other hand, the administration of FA resulted in marked recovery and maintenance of the normal architecture of neurons and neuroglia in the cerebral cortex, hippocampus, and cerebellar cortex. These findings settle the neuroprotective role of FA against DEHP neurotoxicity.

Caspase-3 is a main proapoptotic mediator and marker of irreversible apoptosis (Khalifa et al. 2017). Immunohistochemically, the cerebral cortex, hippocampus, and cerebellar cortex-stained sections of DEHP-treated rats exhibited strong positive caspase-3 immunoexpression. These results agreed with Ma et al. (2015), who mentioned that the cerebral cortex and hippocampus of rats treated with diisononyl phthalate showed a significant increase in caspase-3 expression compared to control rats. In contrast, it has been observed that administration of a low dose of DEHP did not significantly alter the expression levels of hippocampus caspase-3 in postnatally exposed male and female rats (Smith and Holahan 2014). However, the administration of FA significantly decreased caspase-3 immunoexpression in the cerebral cortex, hippocampus, and cerebellar cortex compared to the DEHP group. These results conclude that FA had an antiapoptotic effect.

One of our interesting findings in the current study was the IR spectra retrieved from FTIR analysis. FTIR spectroscopy is a reliable biophysical tool that provides strong insight into macromolecule functional and structural alterations within tissue cells (Sedik and Amer 2022). In our study, we applied FTIR spectroscopy to investigate the neurotoxicity induced by DEHP in different animal groups and evaluate the role of FA to counteract the harmful effects of DEHP. IR spectrum from DEHP intoxicated rats revealed a marked reduction in the area and the intensity of amide I bands **(ν**(C = O) of proteins) around 1654 cm^−1^ and amide II bands (**δ** (N–H) and **ν**(C–N)) of proteins around 1544 cm^−1^ in the brain spectrum than the control group. This finding could signify the destructive effect of the DEHP and lead to a change in the composition of the whole protein pattern. This result agrees with a previous study (Ali et al. 2018). Moreover, a marked reduction in the band areas around 900–1300 cm^−1^ corresponds to the nucleic acid. DEHP also revealed an increase in the band area around 1735 cm^−1^; carbonyl ν(C = O) corresponds to lipids due to the drastic attack of free radicals produced from DEHP intoxication. The N–H stretching of proteins was changed significantly in DEHP-intoxicated rats, leading to distortions in the intramolecular hydrogen bonding in the brain tissue proteins. Our findings aligned with a previous study (Forner-Piquer et al. 2017). Administration of FA showed marked amelioration in the amide I and amide II bands, reducing the elevated band area towards proteins and lipids.

ADMET and toxicity studies were also performed to detect the pharmacokinetic behavior of FA and its toxicity level using the ProTox Online Tool (Banerjee et al. 2018). Firstly. Skin permeability (Kp) is an important issue for improving drug efficacy. Kp of FA is − 2.720 cm/h (less than − 2.5) which predicts that FA has good skin penetrability. The Caco-2 permeability is used as an in vitro model for the human intestinal mucosa to predict the absorption of orally administered drugs. A compound is considered to have a high Caco-2 permeability if it has log Papp values > 0.90 cm/s. FA has log Papp = 0.176, which is less than 0.9 cm/s, so it is predicted that FA has low Caco-2 permeability (Banerjee et al. 2018). Also, the volume of distribution VDss value of the FA =  − 1.367, which is less than − 0.15, so it can be predicted that FA could be distributed by providing an equal blood plasma level. FA can penetrate the BBB moderately as the log BBB value of the FA is − 0.239, which means greater than − 1. Log PS (the permeability of the blood-surface area of the CNS) value of the FA is − 2.612, which is less than − 3 that is unable to penetrate the CNS.

Moreover, FA does not affect or inhibit the CYP2D6 and CYP3A4 enzymes, so it can be predicted that it tends to be metabolized by the P450 enzymes in the body. Moreover, the log CLTOT value of the FA = 0.623 ml/min/kg predicts the renal excretion rate. Finally, FA belongs to category 4, and the AMES and hepatotoxicity prediction suggested its safety (Banerjee et al. 2018).

During drug discovery, the drug-likeness and rule of five proposed by Lipinski (2004) predict that poor absorption is more likely to happen if the drug does not exceed 5 hydrogen bonds (2 in FA) and 10 H-bond acceptors (3 in FA); the molecular weight is greater than 500 (194.186 in FA), the calibrated logarithm *P* value is greater than 5 (1.4986 in FA), and the number of revolving bonds exceeds 15 (3 in FA). Therefore, it is highly favorable for FA to follow Lipinski’s rule of five. Moreover, a docking study of FA was employed for the prediction of binding mode and to understand its inhibition ability on caspase-3 (PDB code 1RHR) (Gadelmawla et al. 2022) and NO synthase (PDB code 4NOS) (Fischmann et al. 1999).

In conclusion, due to its potent antioxidant and antiapoptotic properties, FA could mitigate the neurotoxic effect of DEHP. That could be visualized in improving anxiety-like behavior, spatial working memory, and recognition memory impairment. Furthermore, it could reestablish the brain redox system with increased BDNF levels in the brain tissue. Also, it could ameliorate structure and function changes in lipid, protein, and nucleic acid levels. Consequently, FA could restore brain architecture induced by DEHP. All these findings were confirmed by molecular docking studies on caspase-3 and NO synthase to understand the inhibitory ability of FA.

The limitations of our study are that the plasma and brain concentrations of DEHP are not evaluated. Also, the different antioxidant parameters and proinflammatory cytokines are not measured in different brain regions to correlate the biochemical results with the behavioral data. Therefore, further research is imperative to fill these knowledge gaps.

## Data Availability

Data will be available upon request from the authors.
